# New type of quantum spin Hall insulators in hydrogenated PbSn thin films

**DOI:** 10.1038/srep42410

**Published:** 2017-02-20

**Authors:** Liang Liu, Hongwei Qin, Jifan Hu

**Affiliations:** 1 School of Physics, State Key Laboratory for Crystal Materials, Shandong University, Jinan 250100, China

## Abstract

The realization of a quantum spin Hall (QSH) insulator working at high temperature is of both scientific and technical interest since it supports spin-polarized and dssipationless edge states. Based on first-principle calculations, we predicted that the two-dimensional (2D) binary compound of lead and tin (PbSn) in a buckled honeycomb framework can be tuned into a topological insulator with huge a band gap and structural stability via hydrogenation or growth on special substrates. This heavy-element-based structure is sufficiently ductile to survive the 18 ps molecular dynamics (MD) annealing to 400 K, and the band gap opened by strong spin-orbital-coupling (SOC) is as large as 0.7 eV. These characteristics indicate that hydrogenated PbSn (H-PbSn) is an excellent platform for QSH realization at high temperature.

Topological insulators (TIs) are a new class of matter, that are distinguished with conventional insulators through the insulating bulk and metallic boundary states^1^,^2^,^3^,^4^,^5^. In two dimensional (2D) TIs (also called quantum spin hall insulators), the edge states, which are protected by the time-reversal symmetry, are robust against nonmagnetic scattering. The reason is that the only possible backscattering tunnels are forbidden by quantum mechanics, which enables dissipationless transport edge channels and the QSH effect. Furthermore, the broken of the time-reversal symmetry of QSH insulators by introducing ferromagnetism (FM) can lead to QAH effect which supports the quantized Hall effect without external magnetic fields^6^,^7^,^8^. Therefore, 2D TIs are notably promising for low-energy-consumption electronics applications. Recently, many 2D TIs have been discovered, most of which have a small bulk gap because of the quantum confinement effect^9^,^10^,^11^,^12^,^13^,^14^,^15^,^16^,^17^,^18^,^19^,^20^,^21^. Few large-gap 2D TIs consist of heavy elements^22^,^23^,^24^,^25^,^26^,^27^,^28^,^29^,^30^, the large ion radii and long weak bond cause the poor structural stability at high temperature. Such shortages hinder their realistic applications. Thus, large-gap 2D TIs that work at room temperature are of both scientific and technical interest.

Group-IV materials with honeycomb or buckled honeycomb lattices are notably important for electronic applications including 2D TIs because of their exotic electronic properties^31^,^32^,^33^,^34^. Graphene is the first proposed QSH insulator with a notably tiny gap since carbon atoms are too light to achieve a considerable SOC effect^9^,^35^. In silicene, germanene and stanene, the strength of SOC is sufficiently enhanced and creates a sizable bulk gap^10^. Particularly, chemically functionalized stanene was theoretically reported to have a sizable gap of ~0.3 eV^22^, which is sufficiently larger than the thermal motion energy at room temperature (~26 meV). Most importantly, both of them have been successfully synthesized in experiments. A recent theoretical work predicted that the atomic thin lead films with buckled honeycomb, which are named plumbene, had unexpectedly huge gaps up to ~1.1 eV induced by SOC^30^. However, the bonds in plumbene are too long and too weak, which cause poor thermal and chemical stability^15^, thus the realistic existence and feasibility of plumbene synthesis at finite temperature are still in doubt. Therefore, realizing TIs with large topological nontrivial gap and sufficient thermal stability is a challenge.

In this work, we propose that the hydrogenated lead-tin (H-PbSn) monolayer is a notably stable TI with a large-gap based on first-principle calculations. Strong bonds between lead and tin atoms highly stabilize the structure. In *ab initio* Born-Oppenheimer molecular dynamics (BOMD) simulations, H-PbSn films can maintain the honeycomb framework for 18 ps at high temperatures up to 400 K. The nontrivial topological phase in H-PbSn can be well understood by the model of maximally localized Wannier functions (MLWF). In the conventional Kane-Mele model, the SOC interactions are expressed as the hopping between next-nearest-neighbor sites. However in the MLWF expression, the SOC interactions emerge in the nearest-neighbor hopping because of the special spatial distribution of localized orbits. The giant SOC in this film opens a nontrivial energy gap that exceeds 0.71 eV, which is verified by HSE06 hybrid functional calculations and proven by the explicitly calculation of the Z_2_ invariant. This gap is the largest gap among all discovered stable 2D TIs. Thus, the hydrogen decorated PbSn thin films are feasible and form a new promising platform for technical application and fundamental research.

## Results and Discussion

**Figure 1:**
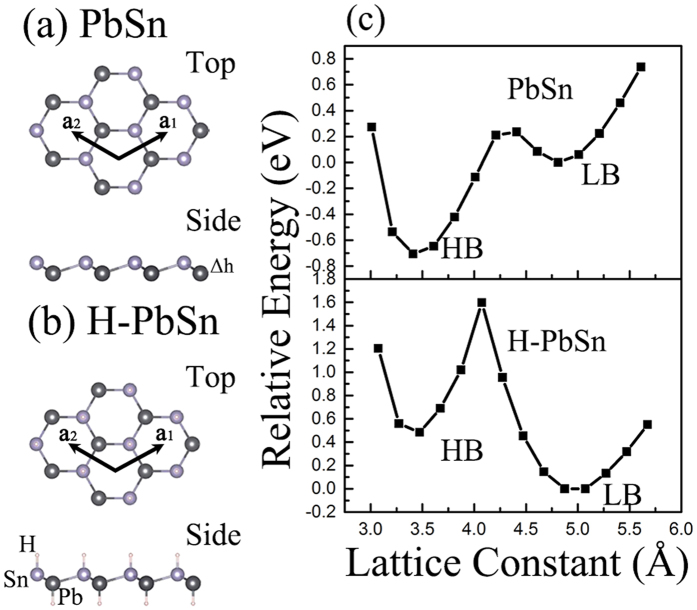
Top and side views of (**a**) pristine PbSn and (**b**) H-PbSn monolayers. The purple, black and small white balls represent the Sn, Pb and H atoms respectively. (**c**) Relative energy (relate to the total energy of the low-buckled structure) versus lattice constant of the PbSn and H-PbSn honeycomb structures. The structures in the left well correspond to smaller lattice constants and larger Δh, and are marked as the high-buckled (HB) state. The ones in the right well correspond to larger lattice constants and smaller Δh and refer to the low-buckled (LB) state.

Figure 1(a) and (b) show the geometrical structures of pristine PbSn and hydrogen decorated PbSn (H-PbSn) films, respectively. The lattice constant dependence of the total energy per unit cell for these two systems is plotted in Fig. 1(c). For pristine PbSn, there is one pair of energy-wells, which include the low-buckled (LB) structure with a larger lattice constant and the high-buckled (HB) structure with a smaller lattice constant. As observed in the upper panel of Fig. 1(c), the HB state is more stable for the pristine PbSn monolayer, in contrast to the plane (PL) geometry of graphene, and LB geometry of silicene, germanene and stanene^22^,^31^,^36^. This difference originates from the large radii of Sn and Pb atoms. The distance between neighboring atoms is too long to have sufficient π-π orbital-coupling and induces high instability. The HB configuration largely hybridizes π orbits with σ orbits and consequently stabilizes the structure and induces the metallic state. This mechanism appears common in 2D honeycomb materials composed of heavy metals^32^. For H-PbSn, the hydrogenation passivates most π orbits and eliminates the instability. The hybridized sp^3^-like σ orbital sufficiently stabilizes the LB lattice and manifests a deep energy well in the LB-range in Fig. 1(c).

**Figure 2:**
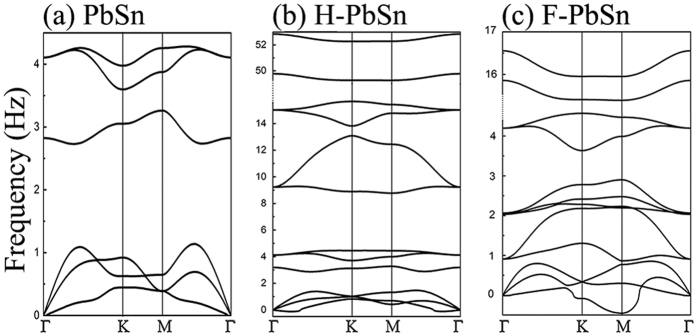
Phonon spectra of (**a**) PbSn, (**b**) H-PbSn and (**c**) F-PbSn along the high symmetric route in the reciprocal space.

**Figure 3:**
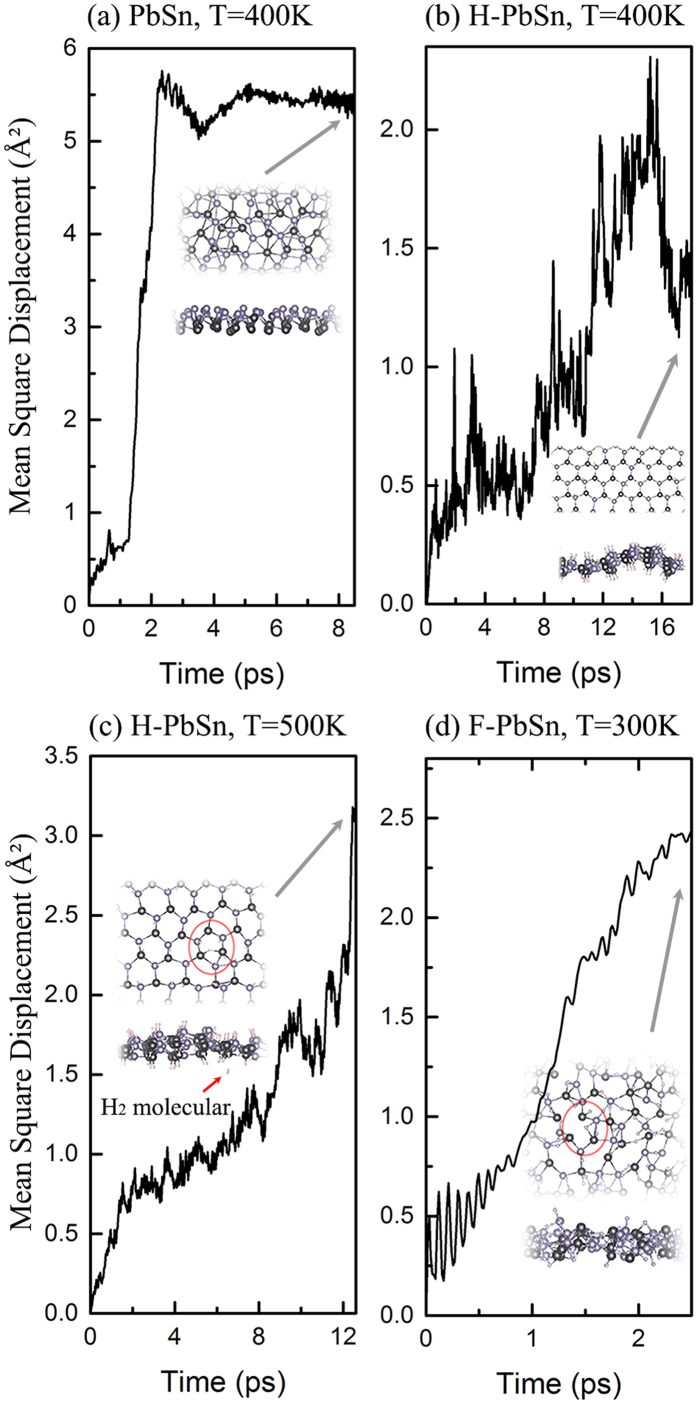
MSD versus time for (**a**) the PbSn monolayer at 400 K (**b**) H-PbSn monolayer at 400 K (**c**) H-PbSn monolayer at 500 K and (**d**) F-PbSn monolayer at 300 K. The snapshots show the top and side views of the final frame of each molecular dynamic simulation. The red circle in (**c**) marks the film fracture and Sn-Sn bond, the red arrow points to the dissolved H_2_ molecules. The red circle in (**d**) marks the film fracture and fluorine atom which bonds to both neighboring Sn atom and Pb atom.

To evaluate the dynamical and thermal stability of the PbSn and H-PbSn monolayers, we performed the phonon calculations and a series of 18 ps BOMD simulations. The phonon spectra are shown in Fig. 2(a) and (b). The negative vibration energies near Г point mean that these structures trend to have a weak distortion over a long range. As observed, the magnitudes of these energies are too small (less than 0.0082 meV for PbSn and less than 0.4136 meV for H-PbSn respectively), we can still believe the kinetic stabilities for both structures. However, as a result of the aforementioned two potential well states, the LB structure of pristine PbSn quickly transforms into the HB structure with large film breakage in MD simulations at 400 K [Fig. 3(a)]. However, the H-PbSn thin films showed excellent ductility and malleability by forming a bending surface to resist against fierce thermal motions, and the honeycomb frameworks survived without fracture up to 400 K [Fig. 3(b)]. Furthermore, the electronic structure calculations using the bending geometrical structure in the last frame of Fig. 3(b) show that the film remained in the topological nontrivial state with a sizable energy gap, which reveals that the nontrivial state is robust against thermal motions. Beyond 500 K, some hydrogen atoms dissolved from the film, and left unsaturated π bonds with instability; then, the 2D hexagonal structures broke down [Fig. 3(c)]. Thus, we confidently believe the existence of H-PbSn at room temperature and its promising feasibility in factual applications.

We also considered other chemical functional groups such as halogen atoms. However, at an instance, the phonon vibration of fluorinated PbSn (F-PbSn) showed a significant negative energy at the **M** point [Fig. 2(c)]. In the MD simulation at 300 K, the honeycomb framework of F-PbSn quickly melted within 2 ps. In the snapshot in Fig. 3(d), some fluorine atoms bonded to two neighboring Pb or Sn ions, weakened the Pb-Sn bond and produced surface fractures. Therefore, we think that F-PbSn cannot work at room temperature. Other halogenated PbSn (Cl-PbSn, Br-PbSn and I-PbSn) films are similar to F-PbSn, and, all are unstable.

**Figure 4:**
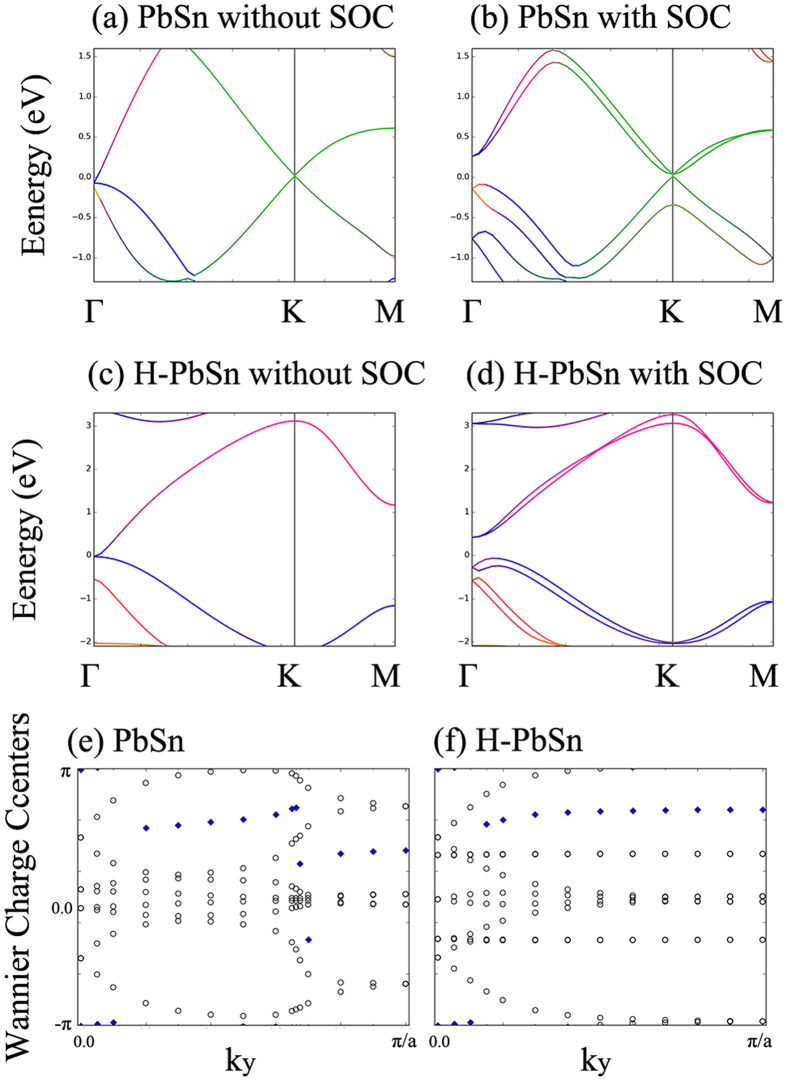
DFT-calculated orbital resolved band structures of (**a**) PbSn without SOC (**b**) PbSn with SOC (**c**) H-PbSn without SOC and (d) H-PbSn with SOC. The red, blue and green lines represent the contribution of the s-orbital, p_x±iy_-orbital and p_z_-orbital proportions in the wave function, respectively. (**e**) and (**f**) show the evolution of WCCs (circles) along the k_x_ direction vs. k_y_ at the k_z_ = 0 plane for PbSn and H-PbSn, respectively. The blue rhombuses are located at the middle of the largest gap.

Figure 4(a) and (b) show the band structures of the pristine PbSn monolayer. Without SOC, similar to graphene, Dirac cones contributed to the p_z_ orbital are formed at the **K** and **-K** point in the momentum space because of the C_3v_ symmetry. Crucially, the gap at the **Г** point is also notably tiny. This result is different from the conventional group-IV atomic thin layers and manifests the energy degeneracy among four p_x±iy_ orbitals^22^,^31^. When the SOC is included, the symmetry of Hamiltonian is lowed, and one must consider more general double group, the lack of the inversion symmetry leads to the lifting of the degeneracy. We can find an indirect band gap of 259 meV at the **Г** and a direct band gap of 30 meV at the **K** or **-K**. We also observe the s-p_x±iy_ orbital reversion at **Г**. Because of the nonequivalent Sn sites and Pb sites, the PbSn honeycomb frameworks lack the space inversion symmetry, and we cannot strictly prove the trivial or nontrivial topological phase simply by examining the parity of occupied bands^37^,^38^. Therefore we calculated the Z_2_ invariant via studying the evolution of the Wannier charge center (WCC) in a half of BZ, following Alexey *et al*.^39^,^40^. In Fig. 4(e), we observe that the gap center of WCC in pristine PbSn jumped twice and both times were over one WCC, the total number of jumped WCCs is even, which proves that Z_2_ = 0. Hence pristine PbSn is a trivial topological insulator, unlike other group-IV monolayers.

The trivial topological phase can be well understood using the orbital analysis and Kane-Mele model^9^. As discussed, there are two types of band reversion over BZ because of the SOC. At **K** and **–K**, similar to stanene and germanene, the occupied states near the Fermi level contribute to the p_z_ orbits which can be described by the two-band Kane-Mele model. For the distinctive band reversion at **Г**, the occupied states near the Fermi level contribute to the p_x±iy_ orbits, which can be described by the four-band Kane-Mele model. Each band reversion can induce a Z_2_ = 1 nontrivial topological phase, but the combination of these two band reversions leads to a Z_2_ = 0 trivial phase since the oddness cancels out exactly.

For the H-PbSn monolayer, the p_z_ orbits are saturated by hydrogenation and move far away from the Fermi level. The degeneracy at **Г**, which is mainly attributed to the four-fold p_x±iy_ orbits, should not be affected by decoration, as observed in Fig. 4(c). The band structure of H-PbSn with SOC is plotted in Fig. 4(d). Degeneracy at the **Г** point is lifted, and the s-p_x±iy_ band reversion emerged, which creates a robust bulk gap of 645 meV. As discussed, we can expect a nontrivial topological phase in H-PbSn, which corresponds to the four-band Kane-Mele model. We also proved the nontrivial topology by the evolution of WCCs, as plotted in Fig. 4(f), where we observe that the gap center jumps over one WCC only one time, which demonstrates that Z_2_ = 1. Hence, the robust energy gaps in H-PbSn films are topological nontrivial gaps. We further confirmed the results using HSE06 hybrid functional calculations and revised value of this nontrivial gap to 710 meV. This huge gap makes H-PbSn films promising 2D TIs that can work at high temperature.

**Figure 5:**
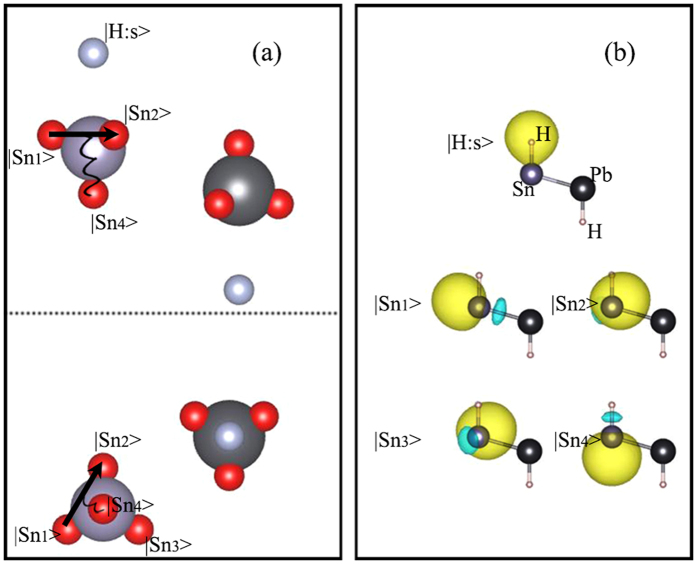
(**a**) Side and top views of Wannier charge centers in real space and mechanism of SOC induced by Coulomb interactions. Purple and black balls represent the sites of Sn and Pb atoms, red balls represent the Wannier charge centers of surrounding orbits of Sn and Pb atoms. Black solid lines imply electron hopping, black curves represent the Coulomb interactions. (**b**) Real space distribution of Wannier wave functions. The yellow and blue isosurfaces represent positive and negative parts of wave functions respectively. The top isosurface is the s orbit of H atom, the lower four isosurfaces are the sp3 hybrid orbits of Sn atom.

To gain a deeper physical understanding of this new 2D topological film, we constructed the maximally localized Wannier functions (MLWF) using DFT-generated parameters^41^. Note, we did not consider the contribution of d orbitals in our study, since there are no transitional metals in our system. In Fig. 5(a) we plotted the center of each localized orbit, which apparently reveals the distinctions from the conventional atomic orbital assumption. Our Wannier orbitals are rather sp3 hybrid orbitals, one can obviously see this characteristic from the real orbitals plotted in Fig. 5(b). For the hybrid orbitals of Sn, we marked them as |Sn_1_>, |Sn_2_>, |Sn_3_> and |Sn_4_>. These four hybrid orbitals located close to each other, which form three-dimensional configuration. This result is essentially different from the 2D Kane-Mele model, and has more degrees of freedom in the hopping progress. First, the sp3 hybrid orbitals are not orthogonal to each other, thus the hopping among the four orbitals is permitted. Second, significantly for this study, each hopping route between two nearest hybrid orbitals is notably close to the nucleus, thus the nearest neighbor hopping will be easily affected by the coulomb fields. In the frame of hopping electron, we can think the hopping electrons experience a magnetic field, and the magnetic field will interact with the spin of electrons, which is exactly the origin of SOC interaction. In general, SOC interactions in the Pauli equation can be expressed as: 
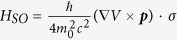
 where ħ is the reduced Planck constant, m_0_ is the mass of free electron, c is the speed of light, V is the coulomb potential of nucleus, **p** is the momentum and **σ** is the Pauli vector. Considering from the configuration of hopping tunnels, we can divide the Hamiltonian into two parts including those parallel with and perpendicular to the plane of film.

For the first part, the gradient of coulomb potential (that is, the coulomb force) is parallel to the plane of film: 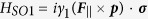. If the hopping tunnel is also parallel to the plane, for example, the hopping between |Sn_1_> and |Sn_2_>, the first part can further expressed as: 
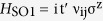
. Anti-symmetric tensor *v*_*ij*_ is a sign factor depending on the direction the electron is traveling. *σ*^z^ is Pauli matrix, only spins pointing z direction survived because the force and momentum are parallel to the plane. For the other hopping tunnels, for example, the hopping between |Sn_1_> and |Sn_4_>, this part is more complex but sufficient small and we do not take it into account. For the second part, the force is perpendicular to the plane thus the Hamiltonian contain the contribution of *σ*^*x*^ and *σ*^*y*^: 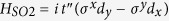.

Finally, we construct our tight-binding model:


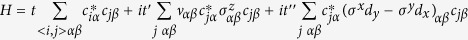


in which c* and c are the creation and annihilation operator for Wannier orbits respectively. The first term is usual spin-independent hopping term, which includes the first and second neighbor hoppings. The indexes i, j represent the atoms and α, β represent the orbitals. Our basis is sp3 orbital, the hybrid orbitals of one atom is not orthogonal to each other thus the hopping between them should be considered. The second and third imagine terms are the effective SOC, we only consider the SOC for hopping among the hybrid orbitals of the same atom.

**Figure 6:**
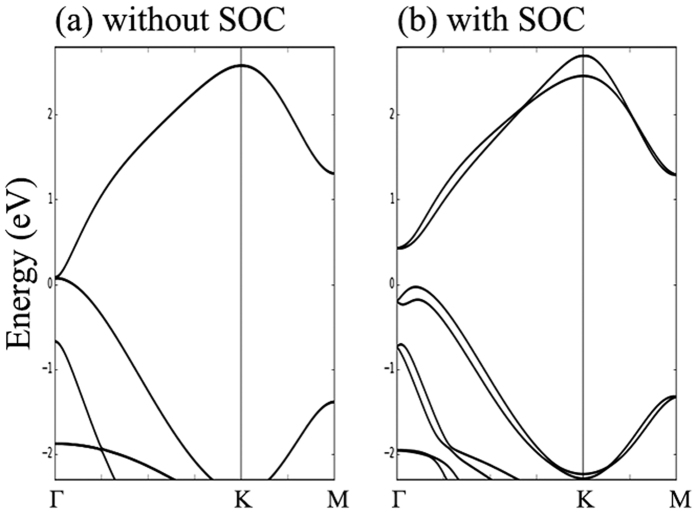
Tight-binding calculated band structures (**a**) w/o SOC (**b**) with SOC.

If we only calculate the first terms, as seen in Fig. 6(a), we obtain a spin-degenerated band structure, particularly at **Г**, where there is an extra degeneration origin from the symmetry. When the SOC term is included, the anti-symmetric terms lift the degeneration and produce an indirect gap. The final band structure calculated by our TB model, which is plotted in Fig. 6(b), perfectly coincides with the DFT result in Fig. 4(d). This result reveals the effectiveness of our model for understanding the topological nature by the hopping between hybrid orbitals.

**Figure 7:**
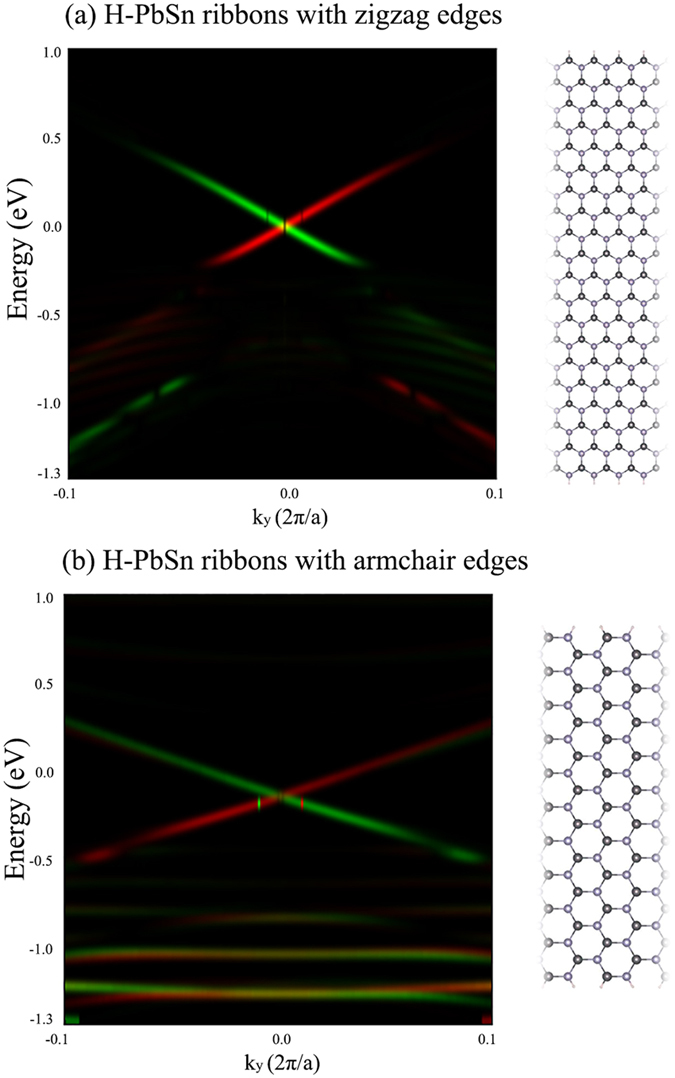
Calculated density of the spin-polarized edge states for H-PbSn nanoribbons with passivated (**a**) zigzag edges and (**b**) armchair edges. The green and red components in each image denote the spin-up and spin-down polarizations respectively. The right panel gives the corresponding nanoribbon models.

The edge state with odd Dirac cones is the most crucial feature of quantum spin Hall insulators. We constructed the H-PbSn nanoribbon models to explore these edge states. The widths of nanoribbons exceeds 6 nm to suppress the intra interactions between opposite sides. The metallic edge states of H-PbSn stripes with zigzag edges and armchair edges are shown in Fig. 7. Both models support gapless edge states, which form Kramer pairs and crossing Fermi level with the single Dirac node at the **Г**, indicating an excellent transport property for various spintronic applications since these metallic states are protected by the time-reversal symmetry and robust against all nonmagnetic scattering. Note that the considered edges of the models are hydrogenated to passivate dangling bonds, which is important to achieve topological edge states. According to our calculations, H-PbSn stripes with bare zigzag edges retain gapless edge states, which are protected by the time-reversal-symmetry, the only difference is the Dirac node locate at the π/a momentum point. However, in the case of H-PbSn stripes with bare armchair edges, however, the metallic edge states are eliminated because of the dangling bonds induce intrinsic magnetism, which break the time-reversal symmetry. According to our calculations, each bare edge has 0.5 μ_B_ magnetic moment, and the neighboring edges trends to ferromagnetic coupling. The energy of ferromagnetic state is lower than anti-ferromagnetic state for 10 meV. Both magnetic states have energetic favor than nonmagnetic state. This characteristic is similar to the graphene nanoribbons and Boron Nitride Nanoribbons^42^,^43^.

Substrates often play crucial roles in the electronic structure of 2D materials, such as driving the thin films that grow on them into nontrivial phases or violating the QSH phases. In general, corresponding to the interaction between the substrate and the thin film that grow on it, there are two types of substrates: weak interacting substrate and strong interacting substrate. In the former case, the films are weakly bonded to the substrate via van der Waals interactions. If grown on the strong interacting substrate, the films are chemically bonded to the substrate commonly with charge transfer.

**Figure 8:**
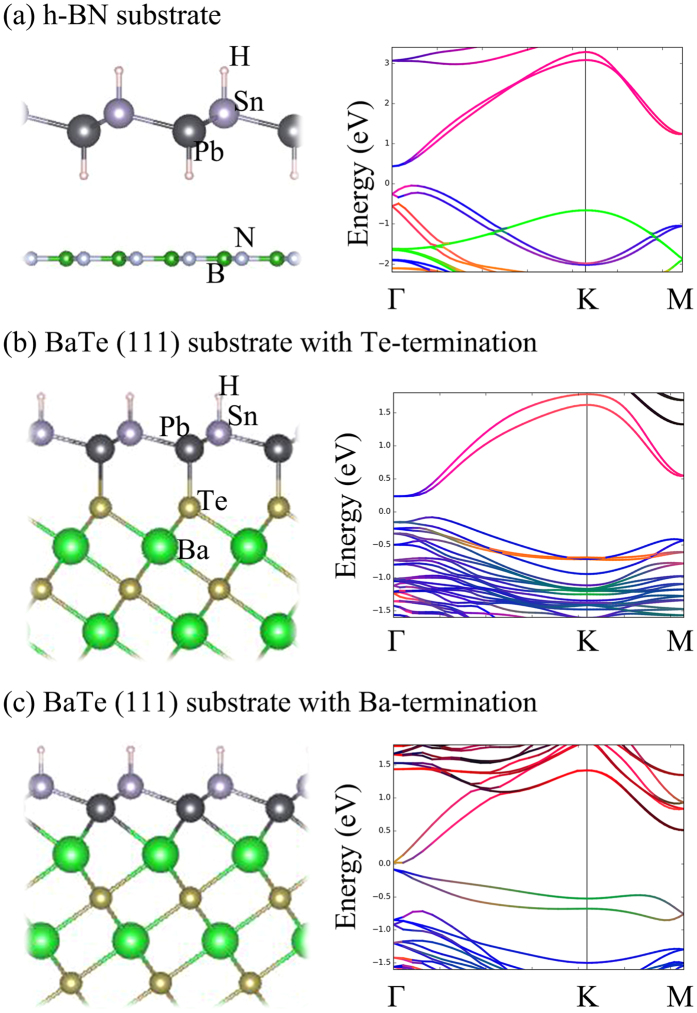
Side views and band structures of (**a**) H-PbSn film that grew on the h-BN substrate (**b**) H-PbSn film that grew on the BaTe (111) surface with Te-termination (**c**) H-PbSn film that grew on the BaTe (111) surface with Ba-termination.

First, let us consider the weak interacting substrates of h-BN due to their large gap and high dielectricity. In addition, h-BN has the identical hexagonal lattice as H-PbSn and the lattice constant of fully relaxed H-PbSn (4.88 Å) is approximately double that of h-BN (5.02 Å), which provides a good environment to grow H-PbSn films. The geometrical structure of H-PbSn that grew on h-BN is shown in Fig. 8(a). Note that the full geometry relaxations were performed under electric dipole correction consideration. We observed the notably long distance between the substrate and the H-PbSn film, which left ample space for hydrogenation. The weak substrate interaction via van der Waals force was too weak to affect the nontrivial topology of H-PbSn, as proven by our Z_2_ invariant calculations.

H-PbSn can also grow on strong interacting substrates. Here we show the (111) surface of BaTe as an instance, since the optimized lattice constant of BaTe (111) is 4.98 Å, which is also close to H-PbSn. Unlike the case of h-BN, BaTe (111) chemically bonded to the H-PbSn film. There are two types of geometrical structures that correspond to the substrate termination: Te-termination [Fig. 8(b)] and Ba-termination [Fig. 8(c)]. In the case of Te-termination, the Pb atoms of the film were located at the top of exactly Te atoms. From the band structure, we can observe that the p_z_ orbitals (green lines) were far away from the Fermi level, which indicates that the dangling π orbits were passivated by substrate. Thus, the BaTe (111) surface with Te-termination served as an orbital filter in the identical manner of the hydrogenation. Our calculations for the Z_2_ invariant also proves the preservation of the nontrivial topological phase in this case. For the Ba-termination case, the Pb atoms were not exactly located at the top of Ba atoms after the full geometrical relaxation. Comparing to the former anion-termination case, the band structures show two extra bands near the Fermi level, and the p_z_ orbital proportion at the **K** indicates that the dangling π bonds were not saturated. The residual p_z_ orbits violated the QSH state of the H-PbSn film because our calculations of Z_2_ had a zero result.

In summary, we have investigated the geometrical and electronic structures of PbSn in the buckled honeycomb structure based on DFT calculations. Hydrogenation can tune this 2D film into nontrivial topological phase, which is confirmed by our Z_2_ invariant calculations and the metallic edge states of nanoribbons. Our MLWF calculations show that the SOC interactions, which induce the nontrivial topological phases, can be understood by the effect of the build-in electric field on the hopping among the sp3 hybrid orbits. The geometrical structure stability at 400 K and the robust nontrivial band gap of ~0.7 eV manifest the promising application of H-PbSn at room-temperature. Furthermore, the large-gap nontrivial topological phases of H-PbSn films were preserved while they grew on an h-BN or BaTe (111) surface with anion-termination. Thus, we believe that the experimental preparation of this 2D TI is highly feasible, which makes it an excellent platform for QSH fundamental studies and electronic applications.

## Methods

Our calculations were performed within the DFT formalism using the generalized gradient approximation (GGA) with Perdew-Burke-Ernzerhof (PBE) as exchange-correlation functional, and the projected augmented wave (PAW) approach as implemented in Vienna ab initio simulation package (VASP) code package^44^,^45^. An energy cutoff of 400 eV was used for the plane-wave expansion of the electronic wave function. Brillouin zones (BZ) were sampled to 8 × 8 × 1 Г-centered meshes for 2D layers and 1 × 8 × 1 meshes for nanoribbons using Monkhorst-Pack method. The energy convergence criteria during full self-consistency in the electronic structure calculations with or without SOC were set at 10^−6^ eV per unit cell. The lattice vectors and positions of the involved atoms were fully relaxed until the max residual force was less than 10^−3^ eV/Å. To avoid spurious interplay interactions, 15 Å vacuum spaces were set in all non-periodic directions of the 2D layers and nanoribbons. The electronic structures were further verified using the Heyd-Scuseria-Ernzerhof (HSE06) hybrid functional which incorporates approximately ten percent nonlocal Hartree-Fock exchange energy in the short range electron-electron interaction region to avoid the inaccuracy of GGA results particularly the underestimation of the band gap^46^,^47^,^48^. For phonon spectra calculations, 3 × 3 2D supercells were used in the finite displacement method in the phonopy code^49^. *Ab initio* BOMD simulations were performed for 15 ps with a time step of 1.5 fs. The NPT ensemble in the Langevin dynamics formalism^50^ were used and all friction coefficients were set to 10.

## Additional Information

**How to cite this article**: Liu, L. *et al*. New type of quantum spin Hall insulators in hydrogenated PbSn thin films. *Sci. Rep.* **7**, 42410; doi: 10.1038/srep42410 (2017).

**Publisher's note:** Springer Nature remains neutral with regard to jurisdictional claims in published maps and institutional affiliations.
